# Entropy-Aware Model Initialization for Effective Exploration in Deep Reinforcement Learning

**DOI:** 10.3390/s22155845

**Published:** 2022-08-04

**Authors:** Sooyoung Jang, Hyung-Il Kim

**Affiliations:** 1 Intelligence Convergence Research Laboratory, Electronics and Telecommunications Research Institute (ETRI), Daejeon 34129, Korea; 2 Artificial Intelligence Research Laboratory, Electronics and Telecommunications Research Institute (ETRI), Daejeon 34129, Korea

**Keywords:** deep reinforcement learning, entropy, model initialization, exploration

## Abstract

Effective exploration is one of the critical factors affecting performance in deep reinforcement learning. Agents acquire data to learn the optimal policy through exploration, and if it is not guaranteed, the data quality deteriorates, which leads to performance degradation. This study investigates the effect of initial entropy, which significantly influences exploration, especially in the early learning stage. The results of this study on tasks with discrete action space show that (1) low initial entropy increases the probability of learning failure, (2) the distributions of initial entropy for various tasks are biased towards low values that inhibit exploration, and (3) the initial entropy for discrete action space varies with both the initial weight and task, making it hard to control. We then devise a simple yet powerful learning strategy to deal with these limitations, namely, entropy-aware model initialization. The proposed algorithm aims to provide a model with high initial entropy to a deep reinforcement learning algorithm for effective exploration. Our experiments showed that the devised learning strategy significantly reduces learning failures and enhances performance, stability, and learning speed.

## 1. Introduction

Reinforcement learning is a commonly used optimization technique for solving sequential decision-making problems [1]. The adoption of deep learning technology to reinforcement learning (so-called deep reinforcement learning (DRL)) has shown successful performance even with high-dimensional observations and action spaces in fields such as robotic control [2,3,4,5,6], gaming [7,8,9], medical [10,11], and financial [12,13] applications. In such a DRL framework, the exploration–exploitation trade-off is a crucial issue that affects the performance of the DRL algorithm [14]. Through exploitation, the agent tries to maximize the current moment’s expected reward, whereas exploration is required to maximize the long-term reward during training [15]. In other words, even if the exploitation that makes the best decision over the current information is successful, the solution obtained by DRL would not be optimal without a number of explorations. Therefore, several studies to encourage exploration are being discussed. Incorporating the entropy term in the reinforcement learning (RL) optimization problem is a representative approach to encourage exploration. The entropy term in the DRL framework represents the stochasticity of the action selection. It is calculated based on the output of the policy. Note that the output of the policy is the action selection probability. The evenly distributed output will yield high entropy. Conversely, if the output is biased, its entropy is low. With the biased output, i.e., low entropy, there is a high probability that the agent cannot perform various actions and repeats only certain actions inhibiting exploration. Therefore, various studies are encouraging high entropy [16,17,18,19,20,21].

In [16], a proximal policy optimization (PPO) algorithm was proposed, in which the entropy bonus term was augmented to ensure sufficient exploration motivated by [22,23]. A soft actor–critic (SAC) DRL algorithm based on the maximum entropy RL framework was proposed in [17], where the entropy term was incorporated to improve exploration by acquiring diverse behaviors in the objective with the expected reward. Ref. [21] also adopted the maximum entropy RL framework as it shows better performance and more robustness. In addition, the authors in [24] proposed a maximum entropy-regularized multi-goal RL, where the entropy was combined with the multi-goal RL objective to encourage the agent to traverse diverse goal states. In [25], maximum entropy was introduced in the multi-agent RL algorithm to improve the training efficiency and guarantee a stronger exploration capability. In addition, a soft policy gradient under the maximum entropy RL framework [26] was devised, and maximum entropy diverse exploration [27] was proposed for learning diverse behaviors. However, these approaches, which consider entropy along with other factors (e.g., reward) in the objective, make the handling of low entropy difficult at model initialization. In [20], the impact of entropy on policy optimization was extensively studied. The authors observed that a more stochastic policy (i.e., a policy with high entropy) improved the performance of the DRL. The authors in [28] analyzed the effect of experimental factors in the DRL framework, where the offset in the standard deviation of actions was reported as an important factor affecting the performance of the DRL. These studies dealt with continuous control tasks, where the initial entropy can be easily controlled by adjusting the standard deviation. To the best of our knowledge, for discrete control tasks, neither any research reporting on the effect of the initial entropy nor a learning strategy exploiting it exists. One of the reasons for this may be the difficulty in controlling the entropy of discrete control tasks. The entropy in a discrete control task is determined by the action selection probability obtained through the rollout procedure, whereas, in a continuous control task, the standard deviation determines the entropy.

To address the abovementioned concerns, we have conducted experimental studies to investigate the effect of initial entropy, focusing on tasks with a discrete action space. Furthermore, based on the experimental observations, we have devised a learning strategy for DRL algorithms, namely entropy-aware model initialization. The contributions of this study can be summarized as follows:

- We reveal a cause of frequent learning failures despite the ease of the tasks. Our investigations show that the model with low initial entropy significantly increases the probability of learning failures, and that the initial entropy is biased towards a low value for various tasks. Moreover, we observe that the initial entropy varies depending on the task and initial weight of the model. These dependencies make it difficult to control the initial entropy of the discrete control tasks;
- We devise entropy-aware model initialization, a simple yet powerful learning strategy that exploits the effect of the initial entropy that we have analyzed. The devised learning strategy repeats the model initialization and entropy measurements until the initial entropy exceeds an entropy threshold. It can be used with any reinforcement learning algorithm because the proposed strategy just provides a well-initialized model to a DRL algorithm. The experimental results show that entropy-aware model initialization significantly reduces learning failures and improves performance, stability, and learning speed.

In Section 2, we present the results of the experimental study on the effect of the initial entropy on DRL performance with discrete control tasks. In Section 3, we describe the devised learning strategy, and discuss the experimental results in Section 4. Finally, we detail the conclusions in Section 5.

## 2. Effect of Initial Entropy in DRL

**Figure 1:**
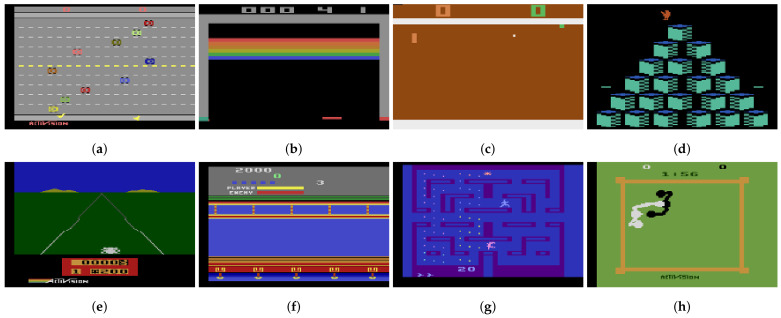
Example of Atari games (with action space size of 3, 4, 6, 6, 9, 14, 18, 18 for Freeway, Breakout, Pong, Qbert, Enduro, KungFuMaster, Alien, Boxing, respectively) used for the experimental study. (**a**) Freeway; (**b**) Breakout; (**c**) Pong; (**d**) Qbert; (**e**) Enduro; (**f**) KungFuMaster; (**g**) Alien; (**h**) Boxing.

To investigate the effect of the initial entropy in the DRL framework, we adopted the policy gradient method (PPO [16]) implementation in RLlib [29]. The network architecture was set to be the same as in [16]. We adopted the Glorot uniform [30], which is the default initializer for Tensorflow [31] and representative RL frameworks such as RLlib, TF-Agents [32], and OpenAI Baselines [33] to initialize the network. Unless otherwise stated, PPO and Glorot uniform are the default settings for the analyses. For this experimental study, we considered eight tasks (please refer to Figure 1) with a discrete action space from the OpenAI Gym [34]. Note that eight tasks (Freeway, Breakout, Pong, Qbert, Enduro, KungFuMaster, Alien, and Boxing) were selected to cover various action space sizes and task difficulties (easy and hard exploration) referring to [35]. Freeway is the game that moves a chicken across the freeway by avoiding oncoming traffic with the action space size of 3. The Breakout game moves a paddle to hit a moving ball to destroy a brick wall, where the action space size is 4. Like the Breakout game, Pong, with the action space size of 6, competes with a computer (left paddle) by controlling the right paddle for rallying the ball, where the paddles move only vertically. In addition, Qbert is a game that moves the cube pyramid and changes the color of the top of the cube and has six action spaces. Next, Enduro is a racing game with nine action spaces aiming to pass an assigned number of cars each day. KungFuMaster is a game in which we fight the enemies we meet on the way to rescue the princess, and it has 14 action spaces. As the game with the most action space, Alien is the game where you destroy aliens’ eggs while avoiding them, and Boxing is the game where we are rewarded by defeating the enemy in the boxing ring. As seen in Figure 1 and the description above, the goals and rules for each of the eight tasks differ. The agent receives the rewards according to the task’s rules in achieving the goals. Therefore, it makes the reward range differ for each task. For example, the range of rewards that an agent can acquire in Pong is from −21 to 21, whereas, in Qbert, it can receive from 0 to more than 15,000. Please refer to [36] for detailed explanations (e.g., description, action types, rewards, and observations) for each game.

**Figure 2:**
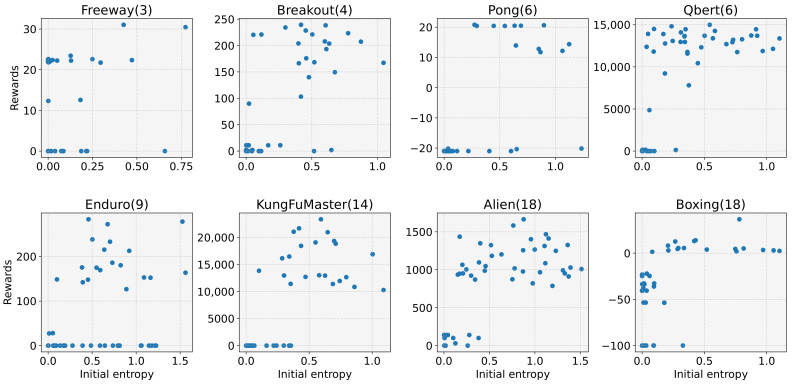
Reward depending on the initial entropy for 8 tasks, where 50 models for each task were generated to investigate the effect of the initial entropy on the performance.

First, we investigated the effect of the initial entropy on performance (i.e., reward). We generated 50 differently initialized models for the experiment and measured the rewards after 3000 training iterations for Freeway, Pong, KungFuMaster, and Boxing, and 5000 training iterations for Breakout, Qbert, Enduro, and Alien. For each iteration, 2048 experiences were collected with 16 workers, and six stochastic gradient descent (SGD) epochs were performed with a learning rate of $2.5\times 10^{-4}$. Figure 2 shows the reward for the initial entropy. We can see that, the lower the initial entropy, the higher the learning failures (e.g., −21 for Pong, 0 for Breakout, and −100 for Boxing). The low initial entropy leads to learning failures by inhibiting exploration. Recall that the entropy is the stochasticity of the action selection probability, and low entropy means the probability is biased towards a specific action. It causes the agent to perform the specific action for every step during the episode with a high probability. Repeating the same action makes exploration difficult. This reminds us of the importance of exploration, particularly during the earlier training stage.

**Figure 3:**
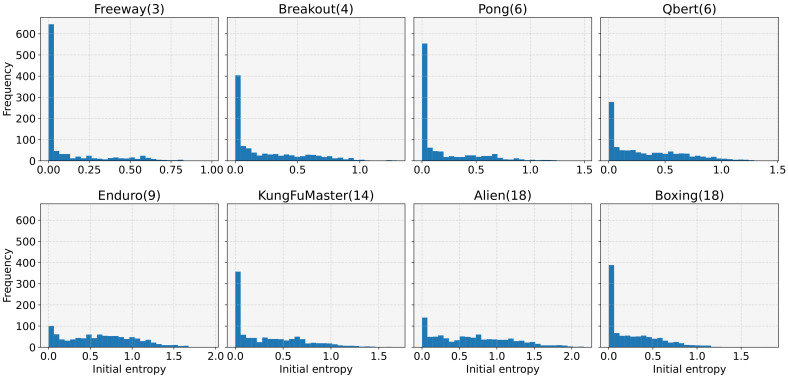
The histograms of the initial entropy for eight tasks. For each task, 1000 models were generated using Glorot uniform initializer with different random seeds.

**Figure 4:**
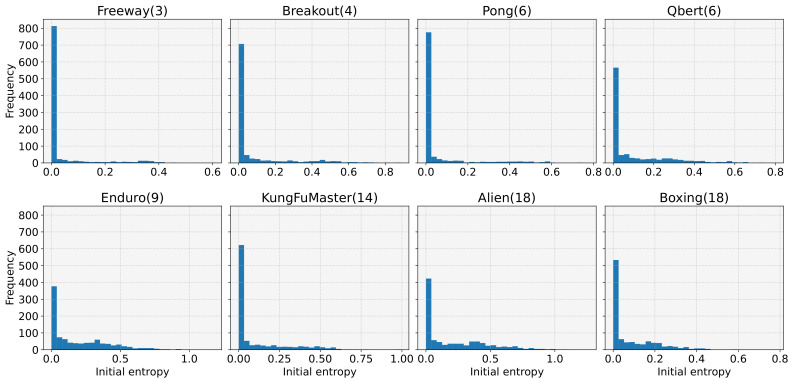
The histograms of the initial entropy for eight tasks. For each task, 1000 models were generated using an orthogonal initializer with different random seeds.

We then investigated the distribution of initial entropy. For this, we generated 1000 models with different random seeds for each of the eight tasks and measured the initial entropy values. Note that the maximum value of the initial entropy is determined by the action space size of the task, for example, 1.099, 1.386, 1.792, 2.197, and 2.890 for action space sizes of 3, 4, 6, 9, and 18, respectively, which are shown in parentheses in Figure 3. From Figure 3, we can see that the initial entropy is biased towards low values, even if the maximum initial entropy value is high, owing to the large action space size. The average initial entropy values were 0.114, 0.246, 0.189, 0.342, 0.636, 0.345, 0.694, and 0.273 for Freeway, Breakout, Pong, Qbert, Enduro, KungFuMaster, Alien, and Boxing, respectively. We performed additional experiments to analyze this tendency on a different network initializer. Specifically, Figure 4 presents the results with an orthogonal initialization technique [37] instead of the Glorot uniform. Nevertheless, we can observe similar trends as in Figure 3. Our experimental findings (i.e., the high probability of learning failures for low initial entropy, and the low biased initial entropy) explain why DRL often fails for tasks with discrete action spaces and why the performance drastically varies for each experiment.

Finally, we investigated the factors affecting the initial entropy. Table 1 and Table 2 show that both the tasks and the initial weight significantly affect the initial entropy. In Table 1 and Table 2, the meaning of seed is a random seed for initializing the neural network. For example, in the first row, Seed 01, of Table 1, the same network, i.e., the same initial weights, are used for measuring the values of Pong and Qbert. The same is true for Alien and Boxing. However, the initial weights of Qbert and Alien differ as the neural network structures differ. Note that the network structure varies according to the size of the action space. For example, for the action space sizes of 6 and 18, the network’s output nodes are 6 and 18, respectively. We can see that the initial entropy varies with the task, even with the same initial weight (e.g., Seed 02’s Alien and Boxing cases in Table 1). In addition, the initial entropy differs according to the initial weight of the model, even with the same task (e.g., Seeds 03 and 04 in Alien cases in Table 1). This is because the input image, which is the observation, differs significantly for each task. These task and model initialization dependencies on initial entropy make it difficult to control the initial entropy.

From the above observations, we conclude that DRL algorithms require models with high initial entropy for successful training, and we need a strategy to generate such models.

## 3. Entropy-Aware Model Initialization

In the previous section, we observed that (1) learning failure frequently occurs with the model with low initial entropy, (2) the initial entropy is biased towards a low value, and (3) even with the same network architecture, the initial entropy greatly varies based on the task and the initial weight of the models. Inspired by the above experimental observations, we propose an entropy-aware model initialization strategy. The learning strategy repeatedly initializes the model until its initial entropy value exceeds the entropy threshold. In other words, the proposed learning strategy encourages DRL algorithms such as PPO [16] to collect a variety of experiences at the initial stage by providing a model with high initial entropy.

Suppose that task (*E*), number of actors (*N*), entropy threshold ($h_{th}$), initializer (*K*), and horizon (*T*) are given. First, we initialize the model ($\pi_{i}$) with *K*. Then, for each *n*-th actor, we perform rollout with the initialized model ($\pi_{i}$) for each time step t∈{1,⋯,T}. Rollout here means the agent interacts with the environment, and, with the rollout, the agent obtains data transitions (i.e., current state, task, reward, and next state) for training. Through the rollout, we store the action selection probabilities ($p_{\pi_{i}}^{\left(n,t\right)}$) for entropy calculation. Note that the action selection probability for the set of actions in action space A (e.g., A={NOOP,FIRE,UP} in the case of Freeway with the action space size of 3) is the softmax of the outputs of $\pi_{i}$. Then, we compute the entropy of the model ($\pi_{i}$) for each actor and the time step as

(1) $$h_{\pi_{i}}^{\left(n,t\right)}=-\sum_{a\in A}p_{\pi_{i}}^{\left(n,t\right)}\left(a\right)\;\log\;p_{\pi_{i}}^{\left(n,t\right)}\left(a\right).$$

Next, the mean entropy (${\hat{h}}_{\pi_{i}}$) of the total action selection probabilities collected from the *N* actors over *T* horizon is computed, which is defined by

(2) $${\hat{h}}_{\pi_{i}}=\frac{1}{NT}\sum_{n=1}^{N}\sum_{t=1}^{T}h_{\pi_{i}}^{\left(n,t\right)}.$$

The mean entropy is compared to the predefined entropy threshold ($h_{th}$). If the mean entropy ${\hat{h}}_{\pi_{i}}$ is larger than the predefined entropy threshold $h_{th}$, then we terminate the entropy-aware model initialization and output the initialized model ($\pi_{init}$) for the DRL algorithm such as PPO. Otherwise, we set the random seed to a different value and repeat the initialization process until ${\hat{h}}_{\pi_{i}}$ exceeds $h_{th}$. The entire entropy-aware model initialization process is summarized in Algorithm 1. Through this learning strategy, the DRL algorithm reduces the probability of learning failure and achieves improved performance and fast convergence to a higher reward (refer to Section 4).
**Algorithm 1:** Entropy-aware model initialization.
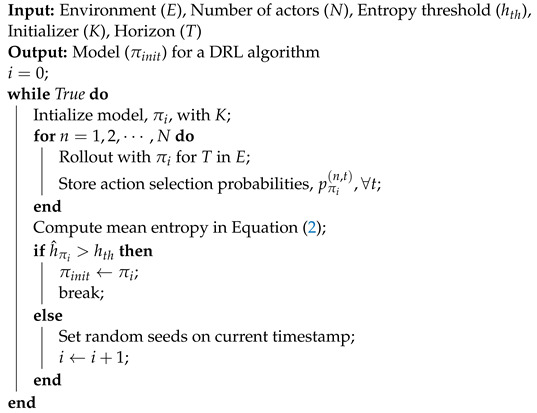


## 4. Experimental Results

In this section, we validate the effectiveness of the proposed learning strategy. For this, we used the experimental settings and tasks described in Section 2. In this experiment, we set the entropy threshold ($h_{th}$) to 0.5.

**Figure 5:**
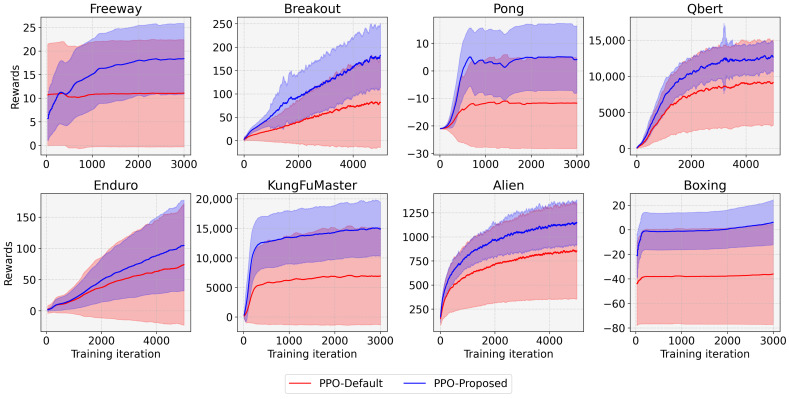
Comparison of the entropy-aware model initialization-based PPO (PPO-Proposed) with the conventional PPO (PPO-Default) for eight tasks.

**Figure 6:**
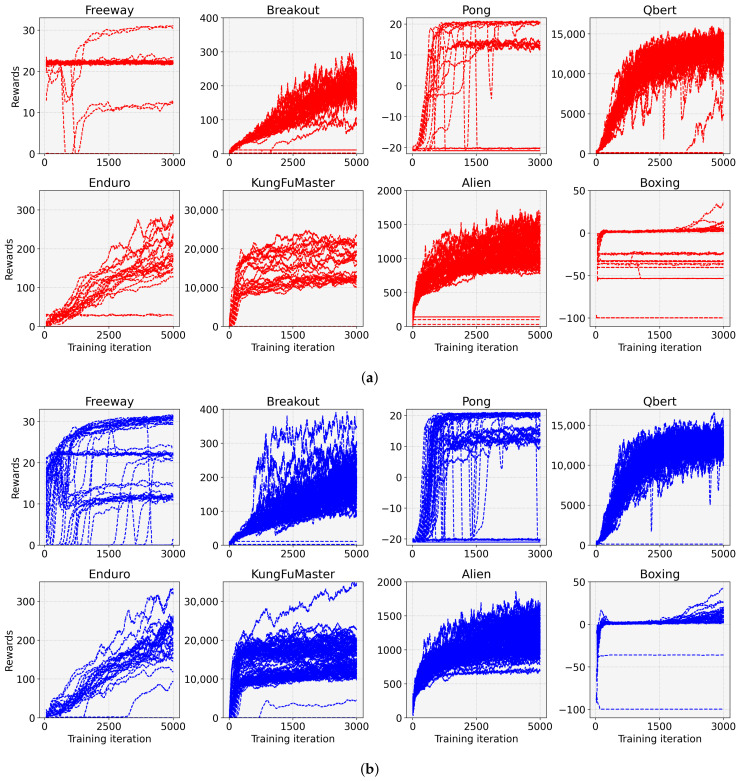
Learning curves for 50 individual experiments of (**a**) the conventional PPO and (**b**) the proposed entropy-aware model initialization-based PPO for 8 tasks. (**a**) default; (**b**) proposed.

To validate the effect of the proposed entropy-aware model initialization, we considered 50 models initialized by different random seeds for each task. Figure 5 shows the rewards according to the training iterations for the eight tasks. In this figure, the red line represents the result for the conventional DRL (without the entropy-aware model initialization) denoted as “Default”, and the blue line denotes the result for the proposed entropy-aware model initialization denoted as “Proposed”. We observed that the DRL with the proposed learning strategy outperformed the conventional DRL for both tasks in four aspects. (1) It restrains the learning failures, e.g., the learning failures for the “Proposed” are 6, 0, 10, 0, 25, 2, 0, and 0, but for the “Default” are 25, 15, 35, 9, 29, 28, 4, and 0, for Freeway, Breakout, Pong, Qbert, Enduro, KungFuMaster, Alien, and Boxing, respectively. (2) It enhances the performance (i.e., average reward in Table 3) by 1.66 for Freeway, 2.22 for Breakout, 2.35 for Pong, 1.39 for Qbert, 1.41 for Enduro, 2.15 for KungFuMaster, 1.34 for Alien, and 2.17 times for Boxing. (3) It reduces the performance variations (i.e., STD of reward in Table 3) with the ratio of 34.22% for Freeway, 29.75% for Breakout, 25.37% for Pong, 65.02% for Qbert, 25.44% for Enduro, 44.63% for KungFuMaster, 53.12% for Alien, and 55.60% for Boxing. (4) It enhances the minimum and maximum rewards as can be seen in Table 3. (5) It enhances the learning speed as can be seen from the slope of the graphs in Figure 5. Figure 6 shows 50 individual learning curves for the above experiments. From the figure, we can easily observe that, by applying the proposed method, more learning curves are biased towards high rewards, and fewer learning failures occur compared to the default.

**Figure 7:**
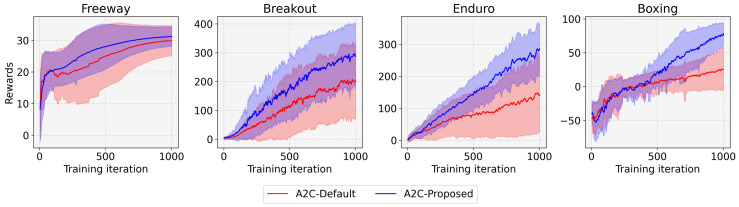
Comparison of the entropy-aware model initialization-based A2C (A2C-Proposed) with the conventional A2C (A2C-Default) for four tasks.

**Figure 8:**
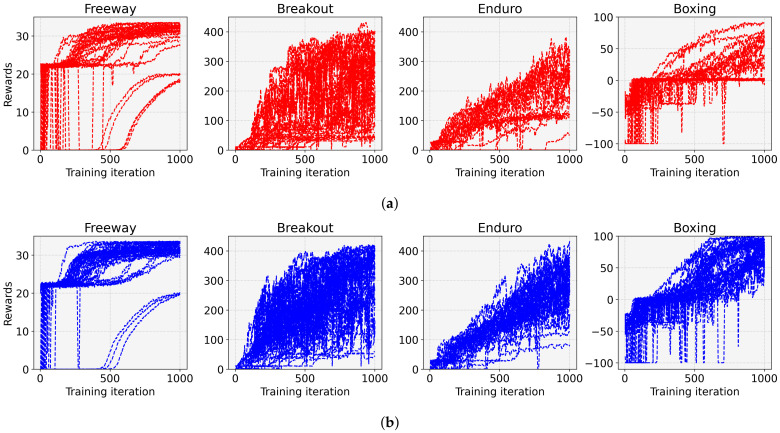
Learning curves for 30 individual experiments of (**a**) the conventional A2C and (**b**) the proposed entropy-aware model initialization-based A2C for four tasks. (**a**) default; (**b**) proposed.

Furthermore, we conducted the experiments with the advantage actor–critic (A2C) [23] instead of PPO for thorough analyses. The results of A2C corresponding to Figure 5 and Figure 6 and in Table 3, of the PPO results are shown in Figure 7 and Figure 8 and in Table 4. We can observe the same phenomena and therefore infer that the proposed algorithm can benefit other DRL algorithms.

Table 5 shows the overhead of the entropy-aware model initialization in terms of the average number and time for repetitive initialization that repeats until the initial entropy becomes larger than the entropy threshold. For the 3000 and 5000 training iterations, the average training times were measured as 4792.75 and 8145.01 s. We can observe that the time overhead of the proposed strategy is negligible compared with the training times. Moreover, the overhead ratio by repetitive initialization in the proposed strategy was reduced because the training time increased as the task became more complex. This is mainly because the overhead of the proposed method is primarily affected by the action space size and initial entropy distribution, and not by the complexity of the task.

**Figure 9:**
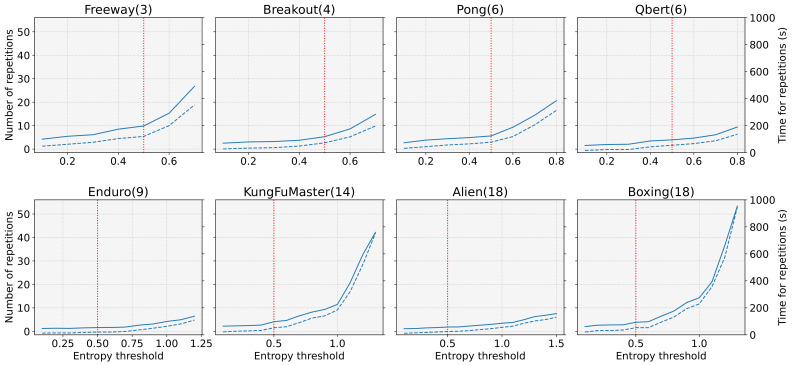
The number (solid line) and time (dashed line) for initialization by the entropy-aware model initialization along the different entropy threshold ($h_{th}$).

Figure 9 presents the number (solid line) and time taken (dashed line) for repetitive initialization along the different entropy thresholds ($h_{th}$). The vertical line in the graph corresponds to when $h_{th}$ is set to 0.5. From Figure 9, we can observe that the time overhead increases according to the entropy threshold; however, the extent of increase is different for each task, the reasons being that (1) different action space sizes of tasks have different maximum initial entropy values, and (2) different tasks have different initial entropy distributions, as shown in Figure 3 in Section 2. In other words, the maximum initial entropy value determines the maximum value of $h_{th}$. The lower the average value of the initial entropy, the faster is the overhead increase. For example, the average initial entropy values of KungFuMaster and Boxing were 0.345 and 0.273, respectively, whereas those of Enduro and Alien were 0.636 and 0.694, respectively. According to Figure 9, we observed that the task (e.g., KungFuMaster) with a low average initial entropy value had a large overhead as the threshold increased. Based on the results in Figure 2 and Figure 9, we set the entropy threshold to 0.5, since the primary purpose of this study is to analyze the effect of initial entropy in DRL and propose a task-independent solution, that is, an entropy-aware model initialization. This value effectively restrains learning failures with tasks of large action space sizes or relatively high initial entropy distribution (e.g., Alien and Boxing) but does not incur much overhead with tasks of small action space sizes or a low-distributed initial entropy (e.g., Freeway and KungFuMaster).

## 5. Conclusions

In this study, we conducted experiments to investigate the effect of initial entropy in the DRL framework, focusing on tasks with discrete action spaces. The critical observation is that models with low initial entropy lead to frequent learning failures, even with easy tasks. These initial entropy values were biased towards low values. Moreover, we observed that the initial entropy varied significantly depending on the task and the initial model weight through experiments under various tasks. Inspired by experimental observations, we devised a learning strategy called entropy-aware model initialization, which repeatedly initializes the model and measures its entropy until the initial entropy exceeds a certain threshold. Its purpose is to improve learning failure, performance, performance variation, and learning speed of a DRL algorithm by providing a well-initialized model to the DRL algorithm. Furthermore, it is practical because it is easy to implement and can be easily applied along with various DRL algorithms without modifying them.

We believe this research can benefit various fields since many applications involve discrete control. Such examples are drone control [5], recommender system [38], and medical CT scans [10]. Moreover, Ref. [39] suggested that discretizing continuous control tasks may improve performance.

It may be a good research direction to propose a neural network initialization technique for deep reinforcement learning with discrete action space. Although many studies proposed initialization techniques for effective deep learning, such as the Glorot uniform and orthogonal, there are few studies on initialization techniques for effective deep reinforcement learning. As can be observed in this paper, the network’s initial state greatly impacts the algorithms’ performance.

## Figures and Tables

**Table 1:** Initial entropy of (Pong, Qbert) pair with action space size 6 and (Alien, Boxing) pair with action space size 18 under different random seeds, where “STD” denotes the standard deviation of the initial entropy values for 10 different random seeds.

	Size of Action Space			
	6		18	
**Task**	**Pong**	**Qbert**	**Alien**	**Boxing**
Seed 01	$1.48\times 10^{-3}$	$4.74\times 10^{-1}$	$3.31\times 10^{-1}$	$2.55\times 10^{-3}$
Seed 02	$9.68\times 10^{-4}$	$8.61\times 10^{-1}$	$8.85\times 10^{-2}$	$8.05\times 10^{-9}$
Seed 03	$9.76\times 10^{-1}$	$2.70\times 10^{-1}$	$9.13\times 10^{-4}$	$1.07\times 10^{-6}$
Seed 04	$7.20\times 10^{-4}$	$2.23\times 10^{-2}$	1.33	$2.05\times 10^{-1}$
Seed 05	$8.04\times 10^{-1}$	$1.58\times 10^{-1}$	$2.25\times 10^{-1}$	$6.35\times 10^{-1}$
Seed 06	$8.98\times 10^{-5}$	$4.68\times 10^{-1}$	$2.76\times 10^{-1}$	$2.11\times 10^{-1}$
Seed 07	$5.64\times 10^{-1}$	$1.58\times 10^{-1}$	$6.05\times 10^{-1}$	$4.39\times 10^{-1}$
Seed 08	$1.18\times 10^{-1}$	$3.42\times 10^{-1}$	$7.95\times 10^{-1}$	$2.88\times 10^{-2}$
Seed 09	$5.73\times 10^{-1}$	$4.34\times 10^{-1}$	$7.28\times 10^{-2}$	$2.30\times 10^{-1}$
Seed 10	$1.73\times 10^{-3}$	$2.79\times 10^{-1}$	$7.91\times 10^{-1}$	$3.79\times 10^{-2}$
STD	$3.85\times 10^{-1}$	$2.33\times 10^{-1}$	$4.22\times 10^{-1}$	$2.15\times 10^{-1}$

**Table 2:** Initial entropy of Freeway, Breakout, Enduro, and KungFuMaster under different random seeds, where “STD” denotes the standard deviation of the initial entropy values for 10 different random seeds.

	Size of Action Space			
	3	4	9	14
**Task**	**Freeway**	**Breakout**	**Enduro**	**KungFuMaster**
Seed 01	$3.78\times 10^{-1}$	$2.95\times 10^{-1}$	1.22	$5.55\times 10^{-4}$
Seed 02	$9.93\times 10^{-14}$	$5.37\times 10^{-12}$	$5.52\times 10^{-1}$	$4.30\times 10^{-8}$
Seed 03	$2.65\times 10^{-1}$	$3.38\times 10^{-1}$	1.52	$4.31\times 10^{-2}$
Seed 04	$9.27\times 10^{-1}$	$8.27\times 10^{-5}$	$2.05\times 10^{-3}$	$1.63\times 10^{-1}$
Seed 05	$9.79\times 10^{-5}$	$6.65\times 10^{-10}$	$9.87\times 10^{-1}$	$3.01\times 10^{-1}$
Seed 06	$2.18\times 10^{-1}$	$9.47\times 10^{-2}$	1.12	$6.29\times 10^{-2}$
Seed 07	$3.75\times 10^{-2}$	$7.13\times 10^{-2}$	1.58	$1.86\times 10^{-4}$
Seed 08	$1.23\times 10^{-1}$	$7.73\times 10^{-1}$	$4.63\times 10^{-1}$	$6.79\times 10^{-8}$
Seed 09	$2.89\times 10^{-3}$	$2.18\times 10^{-2}$	$8.16\times 10^{-1}$	$6.37\times 10^{-1}$
Seed 10	$7.91\times 10^{-4}$	$5.79\times 10^{-1}$	$6.18\times 10^{-1}$	$8.08\times 10^{-2}$
STD	$2.90\times 10^{-1}$	$2.74\times 10^{-1}$	$4.96\times 10^{-1}$	$2.03\times 10^{-1}$

**Table 3:** Statistical results for the experimentation of the entropy-aware model initialization-based PPO and the conventional PPO.

Task	Method	Avg. Reward	STD of Reward	Min Reward	Max Reward
Freeway	Default	11.067	11.369	0	31.04
	Proposed	18.376	7.479	0	31.55
Breakout	Default	81.847	97.855	0	239.27
	Proposed	181.905	68.739	2	348.67
Pong	Default	−11.736	16.507	−21	20.82
	Proposed	4.119	12.319	−21	20.86
Qbert	Default	9141.865	5913.837	0	14,994.75
	Proposed	12,671.130	2068.368	125	15,605.00
Enduro	Default	74.247	97.230	0	283.69
	Proposed	104.804	72.493	0	326.18
KungFuMaster	Default	6926.000	8241.017	0	23,356.00
	Proposed	14,896.011	4562.688	0	34,334.00
Alien	Default	854.550	498.047	0	1665.00
	Proposed	1148.814	233.470	693.60	1665.30
Boxing	Default	−36.100	41.182	−99.94	36.55
	Proposed	6.113	18.284	−99.88	42.10

**Table 4:** Statistical results for the experimentation of the entropy-aware model initialization-based A2C and the conventional A2C.

Task	Method	Avg. Reward	STD of Reward	Min Reward	Max Reward
Freeway	Default	29.839	4.710	18.06	33.41
	Proposed	31.199	3.094	19.59	33.59
Breakout	Default	198.892	131.255	31.00	398.53
	Proposed	287.870	106.686	45.36	412.72
Enduro	Default	141.083	115.656	0	328.90
	Proposed	285.711	79.364	78.26	432.87
Boxing	Default	25.184	30.662	−7.51	90.07
	Proposed	78.129	15.385	48.09	99.39

**Table 5:** The average number and time for initialization, and overhead ratio to the total training time by the proposed entropy-aware model initialization.

Task	Average Number of Initialization (#)	Average Time for Initialization (s)	Time Overhead (%)
Freeway	9.86	119.993	4.000
Breakout	5.30	72.544	1.451
Pong	5.62	77.335	2.578
Qbert	3.94	54.540	1.091
Enduro	1.60	20.516	0.410
KungFuMaster	4.10	52.141	1.738
Alien	1.84	24.536	0.491
Boxing	3.86	53.115	1.771

## Data Availability

Not applicable.

## References

[1] Arulkumaran K., Deisenroth M.P., Brundage M., Bharath A.A. (2017). Deep Reinforcement Learning: A Brief Survey. IEEE Signal Process. Mag..

[2] Yang Z., Merrick K., Jin L., Abbass H.A. (2018). Hierarchical Deep Reinforcement Learning for Continuous Action Control. IEEE Trans. Neural Networks Learn. Syst..

[3] Haarnoja T., Pong V., Zhou A., Dalal M., Abbeel P., Levine S. Composable Deep Reinforcement Learning for Robotic Manipulation. Proceedings of the IEEE International Conference on Robotics and Automation (ICRA).

[4] Lathuilière S., Massé B., Mesejo P., Horaud R. (2019). Neural network based reinforcement learning for audio–visual gaze control in human–robot interaction. Pattern Recognit. Lett..

[5] Jang S., Choi C. (2022). Prioritized Environment Configuration for Drone Control with Deep Reinforcement Learning. Hum. Centric Comput. Inf. Sci..

[6] Zhang Q., Ma X., Yang Y., Li C., Yang J., Liu Y., Liang B. (2021). Learning to Discover Task-Relevant Features for Interpretable Reinforcement Learning. IEEE Robot. Autom. Lett..

[7] Silver D., Schrittwieser J., Simonyan K., Antonoglou I., Huang A., Guez A., Hubert T., Baker L., Lai M., Bolton A. (2017). Mastering the Game of Go without Human Knowledge. Nature.

[8] Patel D., Hazan H., Saunders D.J., Siegelmann H.T., Kozma R. (2019). Improved Robustness of Reinforcement Learning Policies upon Conversion to Spiking Neuronal Network Platforms Applied to Atari Breakout Game. Neural Netw..

[9] Nicholaus I.T., Kang D.K. (2021). Robust experience replay sampling for multi-agent reinforcement learning. Pattern Recognit. Lett..

[10] Ghesu F.C., Georgescu B., Zheng Y., Grbic S., Maier A., Hornegger J., Comaniciu D. (2017). Multi-scale Deep Reinforcement Learning for Real-time 3D-landmark Detection in CT Scans. IEEE Trans. Pattern Anal. Mach. Intell..

[11] Raghu A., Komorowski M., Celi L.A., Szolovits P., Ghassemi M. Continuous state-space models for optimal sepsis treatment: A deep reinforcement learning approach. Proceedings of the Machine Learning for Healthcare Conference.

[12] Zarkias K.S., Passalis N., Tsantekidis A., Tefas A. Deep Reinforcement Learning for Financial Trading using Price Trailing. Proceedings of the IEEE International Conference on Acoustics, Speech and Signal Processing (ICASSP).

[13] Tsantekidis A., Passalis N., Tefas A. (2021). Diversity-driven Knowledge Distillation for Financial Trading using Deep Reinforcement Learning. Neural Netw..

[14] Ishii S., Yoshida W., Yoshimoto J. (2002). Control of Exploitation–Exploration Meta-parameter in Reinforcement Learning. Neural Netw..

[15] Sun S., Wang H., Zhang H., Li M., Xiang M., Luo C., Ren P. (2022). Underwater Image Enhancement with Reinforcement Learning. IEEE J. Ocean. Eng..

[16] Schulman J., Wolski F., Dhariwal P., Radford A., Klimov O. (2017). Proximal Policy Optimization Algorithms. arXiv.

[17] Haarnoja T., Zhou A., Abbeel P., Levine S. Soft Actor-critic: Off-policy Maximum Entropy Deep Reinforcement Learning with a Stochastic Actor. Proceedings of the International Conference on Machine Learning (ICML).

[18] Seo Y., Chen L., Shin J., Lee H., Abbeel P., Lee K. State Entropy Maximization with Random Encoders for Efficient Exploration. Proceedings of the International Conference on Machine Learning (ICML).

[19] Zhang Y., Vuong Q.H., Song K., Gong X.Y., Ross K.W. (2018). Efficient Entropy for Policy Gradient with Multidimensional Action Space. arXiv.

[20] Ahmed Z., Le Roux N., Norouzi M., Schuurmans D. Understanding the Impact of Entropy on Policy Optimization. Proceedings of the International Conference on Machine Learning (ICML).

[21] Chen J., Li S.E., Tomizuka M. (2021). Interpretable End-to-End Urban Autonomous Driving with Latent Deep Reinforcement Learning. IEEE Trans. Intell. Transp. Syst..

[22] Williams R.J. (1992). Simple Statistical Gradient-following Algorithms for Connectionist Reinforcement Learning. Mach. Learn..

[23] Mnih V., Badia A.P., Mirza M., Graves A., Lillicrap T., Harley T., Silver D., Kavukcuoglu K. Asynchronous Methods for Deep Reinforcement Learning. Proceedings of the International Conference on Machine Learning (ICML).

[24] Zhao R., Sun X., Tresp V. Maximum Entropy-regularized Multi-goal Reinforcement Learning. Proceedings of the International Conference on Machine Learning (ICML).

[25] Wang Z., Zhang Y., Yin C., Huang Z. Multi-agent Deep Reinforcement Learning based on Maximum Entropy. Proceedings of the IEEE Advanced Information Management, Communicates, Electronic and Automation Control Conference (IMCEC).

[26] Shi W., Song S., Wu C. (2019). Soft Policy Gradient Method for Maximum Entropy Deep Reinforcement Learning. arXiv.

[27] Cohen A., Yu L., Qiao X., Tong X. (2019). Maximum Entropy Diverse Exploration: Disentangling Maximum Entropy Reinforcement Learning. arXiv.

[28] Andrychowicz M., Raichuk A., Stańczyk P., Orsini M., Girgin S., Marinier R., Hussenot L., Geist M., Pietquin O., Michalski M. What Matters for On-policy Deep Actor-critic Methods? A Large-scale Study. Proceedings of the International Conference on Learning Representations (ICLR).

[29] Liang E., Liaw R., Nishihara R., Moritz P., Fox R., Goldberg K., Gonzalez J., Jordan M., Stoica I. RLlib: Abstractions for Distributed Reinforcement Learning. Proceedings of the International Conference on Machine Learning (ICML).

[30] Glorot X., Bengio Y. Understanding the difficulty of training deep feedforward neural networks. Proceedings of the International Conference on Artificial Intelligence and Statistics (AISTATS).

[31] Abadi M., Agarwal A., Barham P., Brevdo E., Chen Z., Citro C., Corrado G.S., Davis A., Dean J., Devin M. (2015). TensorFlow: Large-Scale Machine Learning on Heterogeneous Systems. https://www.tensorflow.org/.

[32] Guadarrama S., Korattikara A., Ramirez O., Castro P., Holly E., Fishman S., Wang K., Gonina E., Wu N., Kokiopoulou E. (2018). TF-Agents: A library for Reinforcement Learning in TensorFlow. https://github.com/tensorflow/agents.

[33] Dhariwal P., Hesse C., Klimov O., Nichol A., Plappert M., Radford A., Schulman J., Sidor S., Wu Y., Zhokhov P. (2017). OpenAI Baselines. https://github.com/openai/baselines.

[34] Brockman G., Cheung V., Pettersson L., Schneider J., Schulman J., Tang J., Zaremba W. (2016). OpenAI Gym. arXiv.

[35] Bellemare M., Srinivasan S., Ostrovski G., Schaul T., Saxton D., Munos R. Unifying count-based exploration and intrinsic motivation. Proceedings of the Advances in Neural Information Processing Systems (NeurIPS).

[36] (2022). Gym Documentation. https://www.gymlibrary.ml/.

[37] Saxe A.M., McClelland J.L., Ganguli S. Exact Solutions to the Nonlinear Dynamics of Learning in Deep Linear Neural Networks. Proceedings of the International Conference on Learning Representations (ICLR).

[38] Dulac-Arnold G., Evans R., van Hasselt H., Sunehag P., Lillicrap T., Hunt J., Mann T., Weber T., Degris T., Coppin B. (2015). Deep Reinforcement Learning in Large Discrete Action Spaces. arXiv.

[39] Tang Y., Agrawal S. Discretizing Continuous Action Space for On-Policy Optimization. Proceedings of the AAAI Conference on Artificial Intelligence (AAAI).

